# Supplementary material for: Different Infectivity and Transmissibility of H5N8 and H5N1 High Pathogenicity Avian Influenza Viruses Isolated from Chickens in Japan in the 2021/2022 Season
Source: Viruses. 2023 Jan 17;15(2):265. doi: 10.3390/v15020265 (PMC9967648; doi:10.3390/v15020265)
Supplement: Supplementary file 1 [file viruses-15-00265-s001.zip › viruses-2156894 Supplementary Material.pdf]

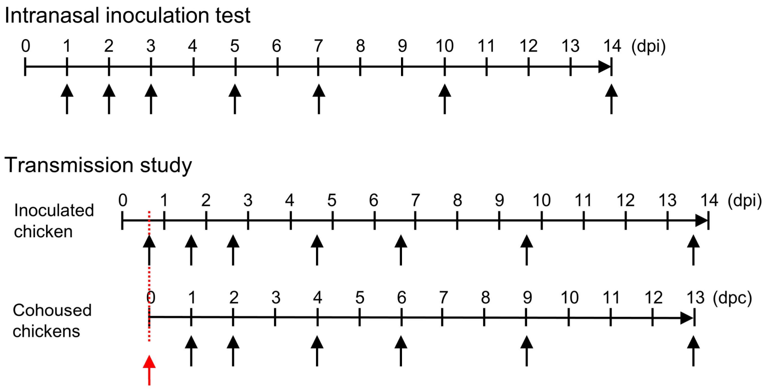

Figure S1. Flowchart of sample collection during animal experiments. In the intranasal inoculation test, at 1, 2, 3, 5, 7, 10, and 14 dpi or at death, tracheal and cloacal swabs were collected. In the transmission study, at 0, 1, 2, 4, 6, 9, and 13 dpc or at death, tracheal and cloacal swabs were collected. Time points for the sample collection were represented by black allows. The time point for the cohabitation in transmission study was shown by a red allow.
